# Phospholipid scramblase-1 regulates innate type 2 inflammation in mouse lungs via CRTH2-dependent mechanisms

**DOI:** 10.1172/JCI169583

**Published:** 2023-08-01

**Authors:** Ashley Hernandez-Gutierrez, Sonoor Majid, Adam Eberle, Ashley Choi, Parand Sorkhdini, Dongqin Yang, Alina Xiaoyu Yang, Carmelissa Norbrun, Chuan Hua He, Chang-min Lee, Chun Geun Lee, Jack A. Elias, Yang Zhou

**Affiliations:** 1 Department of Molecular Microbiology and Immunology and; 2 Department of Internal Medicine, Warren Alpert Medical School, Brown University, Providence, Rhode Island, USA.

**Keywords:** Immunology, Allergy, Asthma, Innate immunity

## Abstract

Exaggerated Type 2 immune responses play critical roles in the pathogenesis of a variety of diseases including asthma, allergy, and pulmonary fibrosis. Recent studies have highlighted the importance of innate type 2 immune responses and innate lymphoid 2 cells (ILC2s) in these disorders. However, the mechanisms that control the development of pulmonary innate type 2 responses (IT2IR) and the recruitment and/or activation of ILC2 cells are poorly understood. In mouse models of pulmonary IT2IR, we demonstrated that phospholipid scramblase-1 (PLSCR1), a type II transmembrane protein that mediates bidirectional and nonspecific translocation of phospholipids between the inner and outer leaflets of the plasma membrane, was a critical regulator of IT2IR in the lung. We further suggested that (a) PLSCR1 bound to and physically interacted with chemoattractant receptor-homologous molecule(CRTH2), which is a G-protein-coupled receptor that is expressed on TH2 cells and on multiple immune cells and is commonly used to identify ILC2 cells, and (b) the effects of PLSCR1 on ILC2 activation and IT2IR were mediated via CRTH2-dependent mechanisms. Overall, our studies demonstrated that PLSCR1 played an essential role in the pathogenesis of ILC2 responses, providing critical insights into biology and disease pathogenesis and identifying targets that can be manipulated in attempts to control IT2IR in chronic diseases such as asthma.

## Introduction

Type 2 immune responses are well documented in normal homeostatic and antipathogen responses in the lung. Exaggerated Type 2 immune responses play critical roles in the pathogenesis of a variety of diseases including asthma, allergy, and pulmonary fibrosis. Early investigations of these disorders focused on adaptive type 2 responses mediated by Th2 lymphocytes. More recent studies have highlighted the importance of innate type 2 immune responses (IT2IR) and innate lymphoid 2 cells (ILC2s) in these disorders. ILC2s are a family of innate immune cells that mirror the phenotypes and functions of adaptive Th2 cells (1). However, the mechanisms that control these responses, particularly the mechanisms that inhibit the development of pulmonary IT2IR and ILC2 cells, are poorly understood. In addition, the degree to which ILC2s can contribute to the chronicity and often life-long responses in diseases like asthma and allergy have not been determined.

Phospholipid scramblase-1 (PLSCR1; Plscr1 in mice) is the most studied member of the phospholipid scramblase protein family. It is a type II transmembrane protein whose main function is the bidirectional and nonspecific translocation (scrambling) of phospholipids between the inner and outer leaflets of the plasma membrane. Scrambling of membrane phospholipids results in the externalization of phosphatidylserine (PS), which acts as a dock for many biological processes including coagulation, apoptosis, and activation of antiinflammatory responses in eukaryotic cells (2). It is well established that PLSCR1 possesses a calcium-binding motif, which is essential for scramblase activity. Studies suggest that phospholipid scramblases are a new class of calcium-binding proteins (3, 4). Other studies have implicated PLSCR1 in signaling (5–7) and demonstrated that PLSCR1 is a receptor for secretory leukocyte protease inhibitor (SLPI), which interacts with CD4 on T cells (8). Surprisingly, the importance of PLSCR1 in normal pulmonary homeostasis and injury and repair has not been defined. In addition, the roles of PLSCR1 in the genesis of IT2IR and ILC2s have not been addressed.

As part of ongoing studies focused on lung immunity, we recently appreciated that PLSCR1 binds to and physically interacts with chemoattractant receptor-homologous molecule (CRTH2), which is expressed on Th2 cells, a known receptor for prostaglandin D2 (PGD2) and Chitinase 3-like-1 (CHI3L1). CRTH2 is expressed on multiple immune cells and is commonly used to identify ILC2 cells in the lung and other organs (9). Recent studies demonstrated that the PGD2-CRTH2 interaction regulates the accumulation of ILC2s and the development of helminth-induced Type 2 pulmonary inflammation (10). Studies reported here demonstrate that the expression of Plscr1 is inhibited by IL-13 at sites of Type 2 inflammation. They also demonstrate that null mutations of Plscr1 significantly augment the baseline expression of the IL-25 receptor, the IL-33 receptor T1/ST2, and the antigen-induced accumulation of lung ILC2 cells. In addition, the effects of Plscr1 on ILC2s and IT2IR are mediated via CRTH2-dependent ILC2 recruitment and activation. These results suggest that PLSCR1 is a critical regulator of IT2IR in the lung and provide critical insights into biology and disease pathogenesis while identifying targets that can be manipulated in attempts to control IT2IR in chronic diseases such as asthma.

## Results

### The expression of Plscr1 is inhibited by IL-13 and Type 2 inflammation.

**Figure 1:**
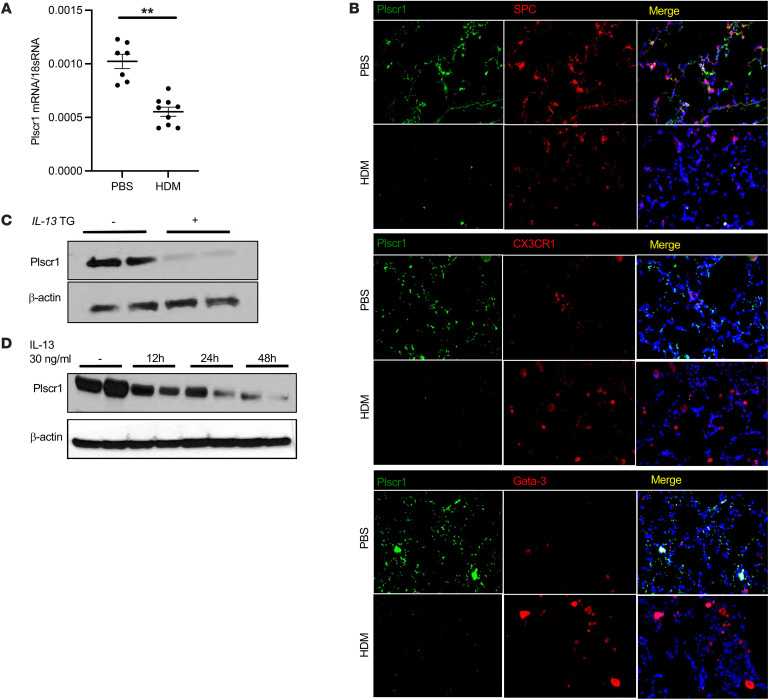
The expression of Plscr1 is inhibited by IL-13 and Type 2 inflammation. (**A**) *WT* mice were subjected to HDM administration, lung Plscr1 mRNA expression was assessed by quantitative real-time PCR. Values are mean± SEM with 7–9 mice in each group. Data was assessed with unpaired Student’s t-test. **P ≤ 0.01. (**B**) Lungs from *WT* mice with or without HDM challenges were sectioned, SPC, CX3CR1, or Gata-3 was labeled with red fluorescence (Alexa Fluor 594) and Plscr1 was labeled with green fluorescence (Alexa Fluor 488). Nuclei are stained with DAPI (blue). Images are representative of 3 mice. (**C**) Whole lung lysates from WT (*IL-13* Tg (–)) and *IL-13* Tg (+) mice were isolated and Plscr1 protein level was then evaluated by Western immunoblot analysis as noted. (**D**) BAL inflammatory cells were isolated from WT mice. Cells were treated with 30 ng/mL IL-13, and Plscr1 protein level was evaluated using Western immunoblot analysis as noted.

To determine the mechanism(s) that contribute to the chronicity of Type 2 inflammation, mice were sensitized and challenged with antigens that are known to induce ILC2/IT2IR and/or Th2 pulmonary responses: house dust mite (HDM) and ovalbumin (OVA). These studies demonstrate that the levels of lung Plscr1 mRNA were decreased after HDM challenges or OVA sensitization and challenge (Figure 1 A and Supplemental Fig.1A). Immunofluorescence staining demonstrated that epithelial cells, CX3CR1^+^ macrophages, and Gata-3^+^ lymphocyte populations had high levels of baseline Plscr1 expression (Figure 1B). With HDM challenges, Plscr1 expression was decreased in epithelial cells, and the macrophages and lymphocytes did not appear to express high levels of Plscr1 (Figure 1B). In addition, the levels of Plscr1 protein were decreased in lungs in which IL-13 was expressed in a transgenic fashion (Figure 1C). Using isolated mouse alveolar inflammatory cells in vitro, we found that Plscr1 was significantly inhibited in cells treated with recombinant (r) IL-13 (Figure 1D). These findings demonstrate that the expression of Plscr1 is inhibited by Type 2 inflammation and IL-13.

### Null mutation of Plscr1 augments allergen-induced type 2 immune responses in the lung.

**Figure 2:**
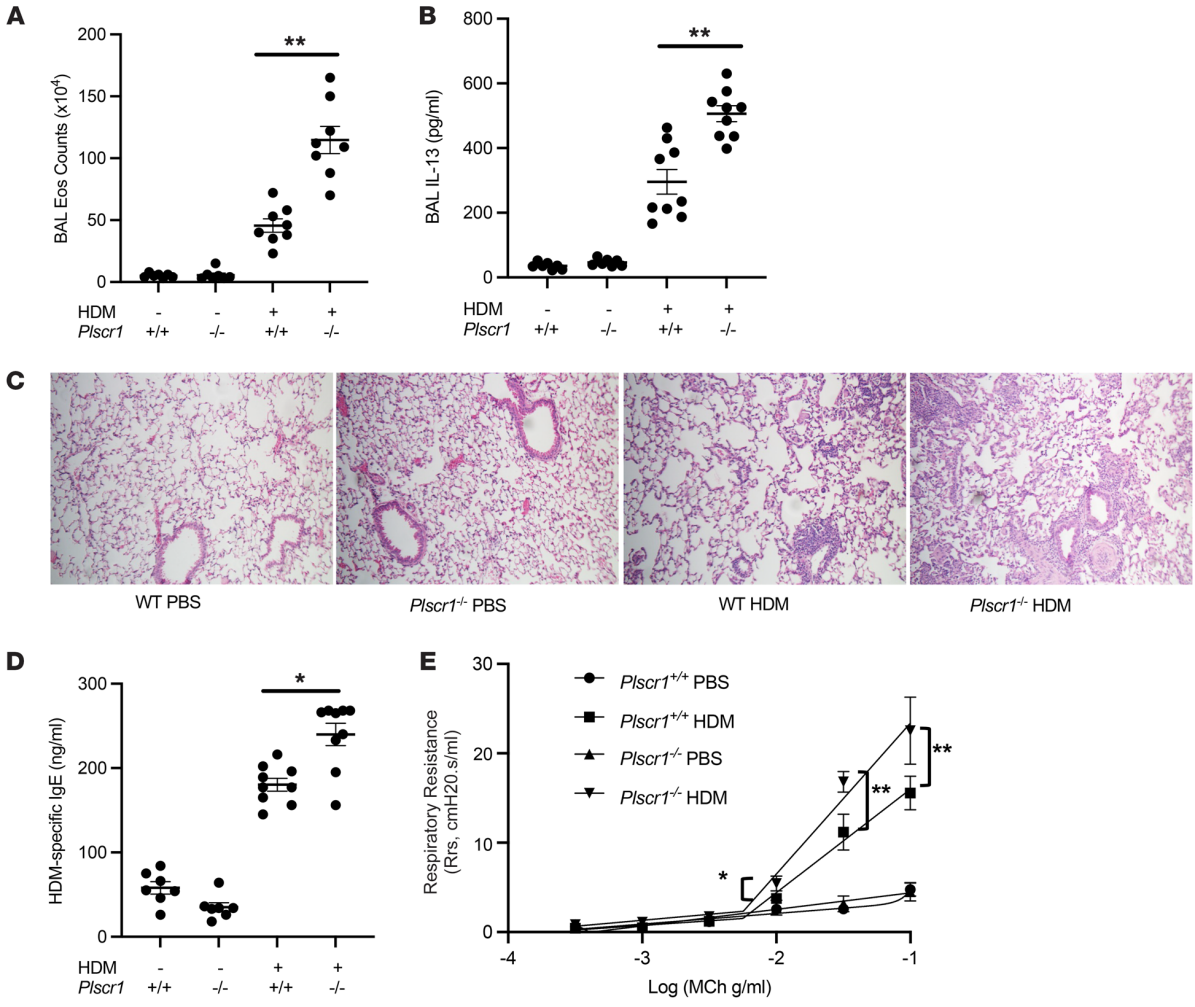
Null mutations of Plscr1 significantly augment antigen-induced type 2 immune responses. *WT* and *Plscr1*^–/–^ mice were subjected to HDM administration, (**A**) BAL eosinophil (Eos) counts were assessed by Diff-Quik staining, and (**B**) BAL IL-13 levels were quantitated by ELISA. (**C**) H&E staining of WT and *Plscr1*^–/–^ mice with and without HDM administration. (**D**) Serum HDM-specific IgE levels were measured by ELISA. (**E**) and total respiratory resistance in response to Methacholine was measured with Flexivent. Values are mean± SEM with 7–9 mice in each group. Comparisons between groups were conducted by 2-way ANOVA with Bonferroni’s posthoc test. *P ≤ 0.05,**P ≤ 0.01.

To define the biologic roles of Plscr1 under Type 2 inflammatory conditions, Plscr1-null mice were generated, and the phenotypes of WT and Plscr1-null mice (*Plscr1^–/–^*) were compared at baseline and after HDM challenge. Mice were challenged with HDM 3 times a week for 3 weeks without prior sensitization to induce an innate immune-dominant inflammatory response in the lung with minimal involvement of T cell-mediated adaptive immunity (11, 12). We found that eosinophil infiltration and IL-13 levels were augmented in Plscr1-null mice (Figure 2, A and B) and the lungs of Plscr1-null mice exhibited exaggerated Type 2 immunopathology (Figure 2C). In addition, HDM-specific IgE levels in the serum and methacholine responsiveness were elevated in Plscr1-null mice compared with WT mice after HDM challenges (Figure 2, D and E). Consistent with these results, using the OVA sensitization and challenge model, which involves both innate and adaptive Type 2 immune responses, similar results were observed, in that *Plscr1^–/–^* mice had increased levels of BAL inflammation, eosinophilia, OVA-specific IgE concentration, and IL-13 secretion (Supplemental Figure 1; supplemental material available online with this article; https://doi.org/10.1172/JCI169583DS1). These studies demonstrate that null mutations of Plscr1 augment antigen-induced Type 2 inflammatory responses.

### Null mutation of Plscr1 augments HDM-induced accumulation of ILC2 cells and innate type 2 immune responses in the lung.

**Figure 3:**
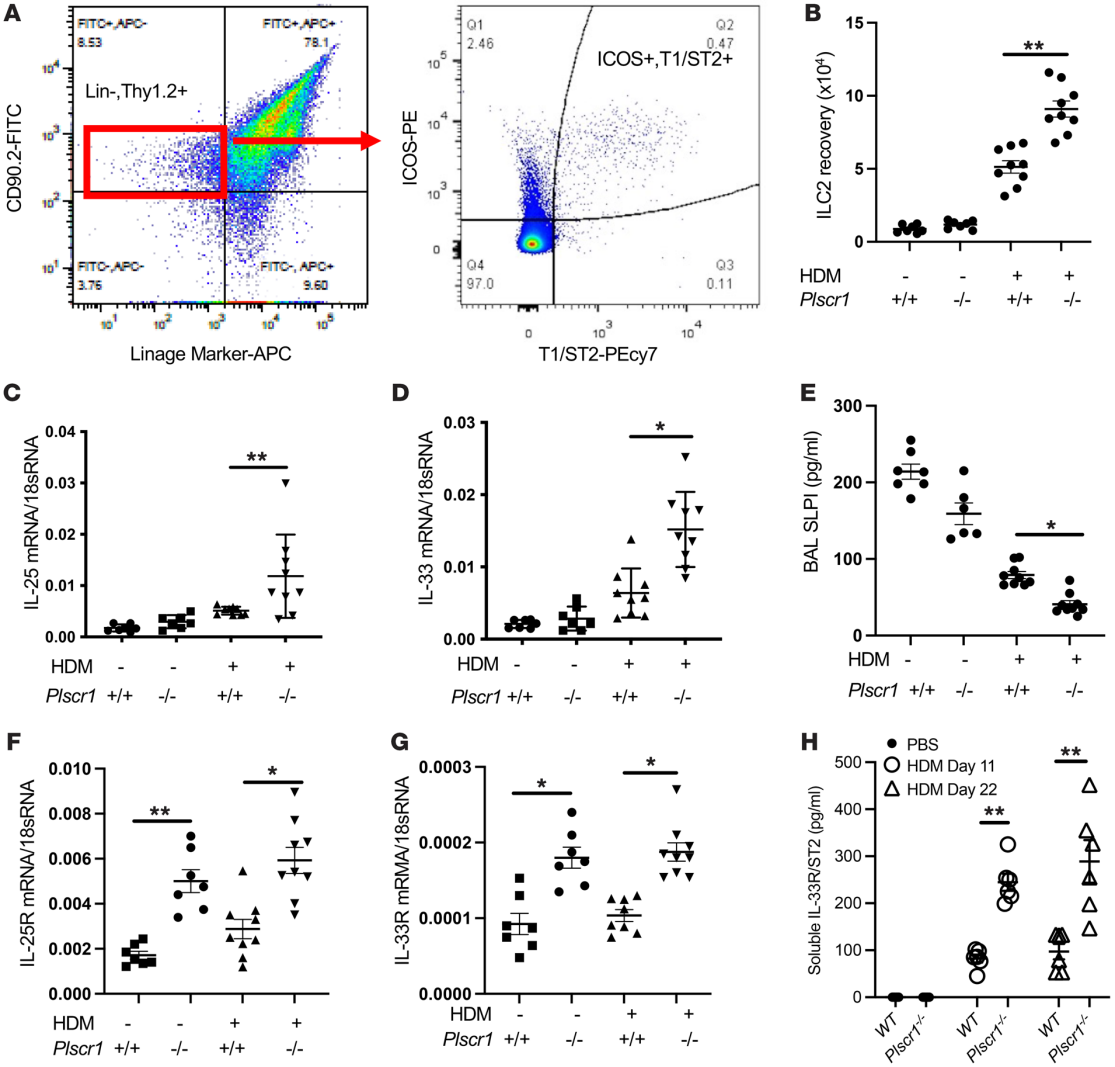
Innate type 2 immune responses are exaggerated in Plscr1-null mice. (**A**) Gating strategy for ILC2 cells in mouse lung. ILC2 are defined as Lin-Thy1.2^+^ICOS^+^T1/ST2^+^ cells. (**B**) *WT* and *Plscr1*^–/–^ mice were subjected to HDM administration (25 μg HDM 3 times a week for 3 weeks), and lung ILC2 recovery was assessed by flow cytometry. (**C** and **D**) Whole lung mRNA was extracted and IL-25 mRNA (**C**) and IL-33 mRNA (**D**) were assessed by RT-PCR. (**E**) BAL SLPI levels were measured by ELISA. (**F** and **G**) ILC2 cells were isolated as described in methods. mRNA was extracted from primary ILC2s; IL-25R mRNA (**F**) and IL-33R mRNA (**G**) were assessed by RT-PCR. (**H**) BAL soluble IL-33R/ST2 levels were measured by ELISA. Values are mean± SEM with 7–9 mice in each group. Comparisons between groups were conducted by 2-way ANOVA with Bonferroni’s posthoc test. *P ≤ 0.05, **P ≤ 0.01.

Because consecutive HDM treatments trigger a primarily innate-dominant ILC2-mediated type 2 immune response, we next focused on using this model to determine if a null mutation of Plscr1 alters the innate immune responses after HDM challenges. We examined ILC2 cell accumulation in the lung by comparing the numbers of linage-Thy1.2^+^ICOS^+^T1/ST2^+^ cells in *WT* and *Plscr1^–/–^* mice by flow cytometry (Figure 3A). In WT mice, HDM modestly increased ILC2 cell accumulation (Figure 3B). Interestingly, after similar treatments, the numbers of ILC2 cells in Plscr1-null mice exceeded those in WT animals (Figure 3B), suggesting that the null mutation of Plscr1 augments innate type 2 immune responses in the lung via ILC2-dependent mechanisms.

To explore the mechanisms that underlie the increased ILC2s and exaggerated IT2IR in Plscr1-null mice, we examined the expression of IL-25 and IL-33 and their receptors, cytokine pathways known to recruit and activate ILC2s. We found that IL-25 and IL-33 expression levels were higher in Plscr1-null mice after HDM challenge (Figure 3, C and D). SLPI is an antiinflammatory protein found in large quantities in bronchial fluids and other mucosal surfaces and fluids (13–15). It is known to interact with Plscr1 (8) and low levels of SLPI are associated with severe type 2 immune responses in mice and humans (16, 17). We found that the levels of SLPI were modestly decreased after HDM challenge in WT mice and were further decreased in Plscr1-null animals (Figure 3E), suggesting that SLPI inhibition may contribute to the exaggerated IT2IR in Plscr1-null mice. We then isolated ILC2s by flow sorting and examined the expression of IL-25 and IL-33 receptors. We found that, at baseline, the expression of IL-25R and IL-33R/ST2 on ILC2 cells from Plscr1-null mice was greater than the levels on ILC2s from *WT* controls. This suggests that pulmonary ILC2s from Plscr1-null mice are primed to be activated (Figure 3, F and G). Consistently, soluble IL-33R/ST2 levels are higher in the BAL fluid of Plscr1-null mice after HDM challenges (Figure 3H).

### ILC2s contribute to exaggerated type 2 immune responses in Plscr1-null mice.

**Figure 4:**
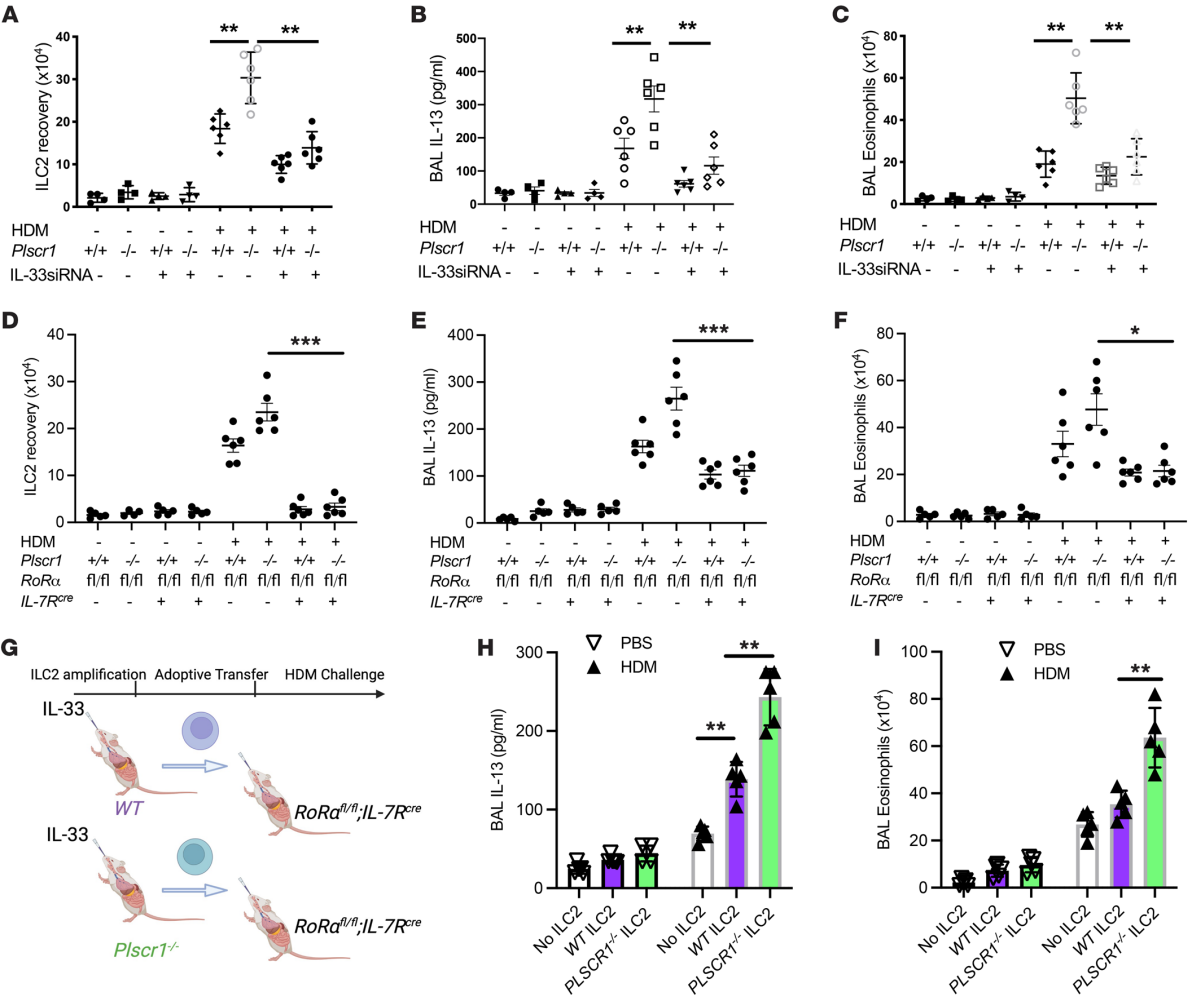
ILC2s contribute to exaggerated type 2 immune responses in Plscr1-null mice. *WT* and *Plscr1*^–/–^ mice were subjected to HDM administration (25 μg HDM 3 times a week for 3 weeks). In **A**–**C**, mice were treated with IL-33 siRNA (every other day, 3 nmol/mouse) or its scrambled control. In (**D**–**F**), *Plscr1*^–/–^ mice were bred with *RoR*α*^fl/fl^* mice and *IL-7R^cre^* mice. (**A** and **D**) Lung ILC2 recovery was assessed by flow cytometry. (**B** and **E**) BAL IL-13 levels were measured by ELISA. (**C** and **F**) BAL eosinophil recovery was assessed. (**G**) *WT* and Plscr1^–/–^ mice were treated with 1 μg IL-33 i.n. daily for 5 consecutive days to allow in vivo ILC2 amplification. 5×10^5^ ILC2s were inoculated i.n. to *RoR*α*^fl/fl^/IL-7R^cre^* mice. Mice were challenged with HDM 1 day after ILC2 adoptive transfer. (**H**) BAL IL-13 levels were measured by ELISA. (**I**) BAL eosinophil recovery was assessed. Values are mean± SEM with 4–7 mice in each group. Comparisons between groups were conducted by 2-way ANOVA with Bonferroni’s posthoc test. *P ≤ 0.05, **P ≤ 0.01. ***P ≤ 0.001.

Studies were next undertaken to define the role(s) of ILC2s in the exaggerated responses in Plscr1-null mice. IL-33 is a major cytokine that is secreted by lung epithelial cells and activates ILC2s (18, 19). We thus used IL-33 siRNA to knockdown the expression of IL-33 and ILC2 activation in vivo. We found that, compared with scrambled siRNA–treated mice, the exaggerated ILC2 accumulation, IL-13 production, and eosinophil accumulation in HDM sensitized and challenged Plscr1-null mice were significantly decreased by the treatment with intranasal IL-33 siRNA (Figure 4, A–C). This highlighted the IL-33 dependence and ILC2 activation of the exaggerated IT2IR in Plscr1-null animals. Alternatively, *RoR*α*^fl/fl^/IL-7R^cre^* mice, in which ILC2s are depleted (20–22), were bred with Plscr1-null mice to examine the specific roles of ILC2s in this model. Our result demonstrated that *Plscr1^–/–^RoR*α*^fl/fl^/IL-7R^cre^* mice lost the majority of the ILC2 population in the lung (Figure 4D), and HDM-induced IL-13 production and eosinophil accumulation were significantly diminished in these mice (Figure 4, E and F). Finally, adoptive transfer experiments were performed to determine if specific loss of Plscr1 on ILC2s mirror this phenotype in *Plscr1^–/–^* mice. We adoptively transferred in vivo amplified ILC2s from IL-33-stimulated *WT* and *Plscr1^–/–^* mice into ILC2-deficient *RoR*α*^fl/fl^/IL-7R^cre^* mice (Figure 4G). We found that HDM-challenged *RoR*α*^fl/fl^/IL-7R^cre^* mice receiving ILC2s from *Plscr1^–/–^* mice developed exaggerated type 2 immune responses compared with mice receiving *WT* ILC2s (Figure 4, H and I). Overall, these studies demonstrated that activated ILC2s contributed to exaggerated type 2 immune responses in Plscr1-null mice.

### PLSCR1 interacts with CRTH2 in vitro and in vivo.

**Figure 5:**
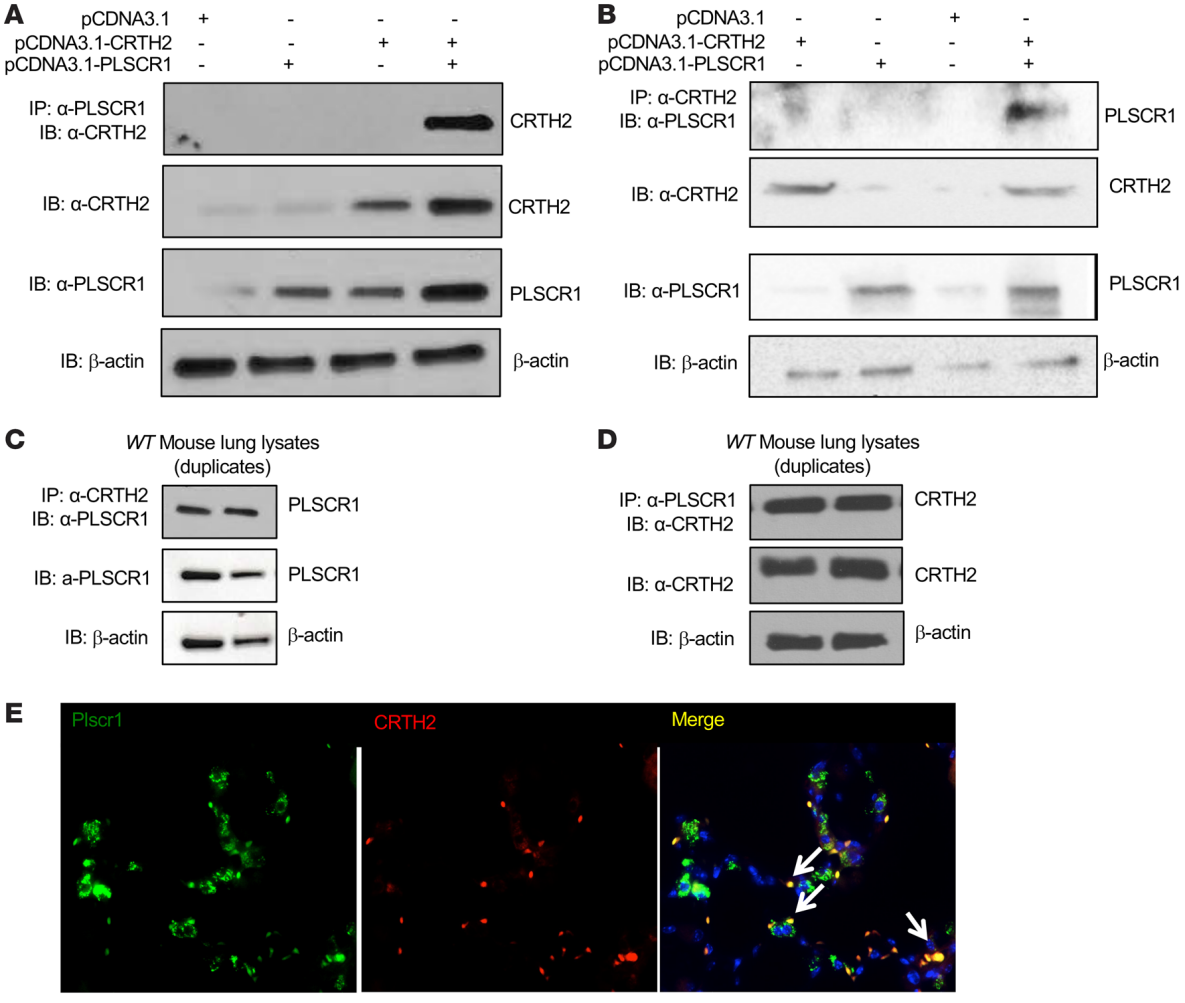
Plscr1 interacts with CRTH2 in vitro and in vivo. (**A** and **B**) HEK293 cells were transfected with CRTH2 and PLSCR1 plasmids and the cell lysates were subjected to either co-IP with α-CRTH2 antibody and immunoblot (IB) with α-PLSCR1, or the opposite. Individual IBs with α-CRTH2 and α-PLSCR1 were also included. (**C** and **D**) Mouse lung protein lysates were subjected to either co-IP with α-CRTH2 antibody and IB with α-PLSCR1, or the opposite. Individual IBs with α-PLSCR1 and α-CRTH2 were also included. (**E**) Lungs from *WT* mice were sectioned, CRTH2 was labeled with red fluorescence (Alexa Fluor 594) and Plscr1 was labeled with green fluorescence (Alexa Fluor 488). Colocalization of CRTH2 and Plscr1 is indicated by arrows. Nuclei are stained with DAPI (blue). Images are representative of 3 mice.

CRTH2, a G-protein-coupled receptor known for binding with PGD2, plays an important role in the pathogenesis of allergic Type 2 inflammation in the lung (23, 24). The expression of CRTH2 is commonly used to identify ILC2s in the lung (25–27). PGD2-CRTH2 interaction regulates the accumulation of ILC2s and Type 2 inflammation, suggesting that CRTH2 plays an important role in IT2IR in the lung. The interactions between PLSCR1 and CRTH2 were verified by cotransfection/coimmunoprecipitation (Co-IP) assays. These evaluations demonstrated that PLSCR1 and CRTH2 physically bind to one another because the immunoprecipitation of one always brought down the other (Figure 5, A–D). IHC evaluations of lungs from *WT* mice demonstrated that Plscr1 and CRTH2 frequently colocalized in these tissues (Figure 5E). The interaction between PLSCR1 and CRTH2 became weaker under Type 2 inflammatory conditions in vivo in *IL-13* Tg lungs (Supplemental Figure 2), most likely because the expression of Plscr1 was lower. These results led us to the hypothesis that CRTH2, and potentially its expression on ILC2s, may regulate Plscr1-mediated ILC2 activation and Type 2 immune responses.

### The effects of Plscr1 on ILC2 and IT2IR are mediated via CRTH2.

**Figure 6:**
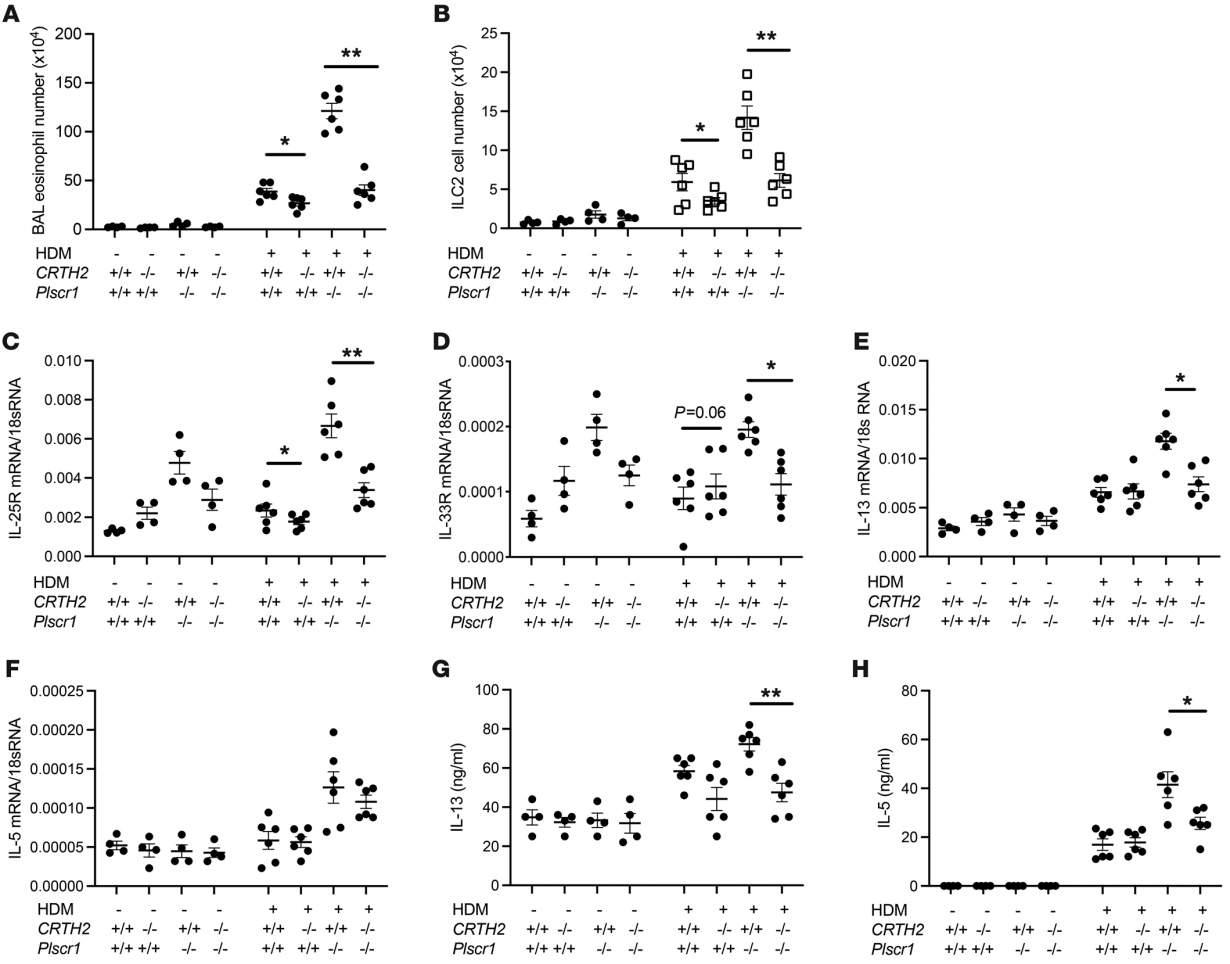
The effects of Plscr1 on ILC2 and IT2IR are mediated via CRTH2. *WT,CRTH2^–/–^*, *Plscr1*^–/–^, and *CRTH2*^–/–^*Plscr1*^–/–^ mice were subjected to HDM administration. (**A**) BAL eosinophil counts were assessed by Diff-Quik staining. (**B**) Lung ILC2 recovery was assessed by flow cytometry. (**C** and **D**) ILC2s were sorted and mRNA was extracted from ILC2s, and IL-25R (**C**) and IL-33R (**D**) mRNA were assessed by RT-PCR. (**E** and **F**) ILC2 were sorted and mRNA was extracted from ILC2s, and IL-13 (**E**) and IL-5 (**F**) mRNA were assessed by RT-PCR. (**G** and **H**) ILC2 were sorted and cultured for 2 days, and IL-13 (**G**) and IL-5 (**H**) protein levels were assessed by ELISA. Values are mean± SEM with a minimum of 4 mice in HDM group. Comparisons between groups were conducted by 2-way ANOVA with Bonferroni’s posthoc test. *P ≤ 0.05,**P ≤ 0.01.

To evaluate the role of CRTH2 in exaggerated ILC2 cell accumulation and IT2IR in *Plscr1^–/–^* mice, we generated *Plscr1^–/–^CRTH2^–/–^* double mutant mice. We next compared HDM-induced ILC2 accumulation and Type 2 inflammation (eosinophil accumulation) in WT mice, Plscr1-null mice, CRTH2-null mice, and *Plscr1^–/–^CRTH2^–/–^* double–null mutant animals. Consistent with our previous results, when compared with the WT controls, HDM-challenged *Plscr1^–/–^* mice manifested exaggerated levels of Type 2 inflammation and ILC2 accumulation (Figure 6, A and B). Importantly, the exaggerated HDM-induced Type 2 inflammation in *Plscr1^–/–^* mice was significantly decreased in the absence of CRTH2 (Figure 6, A and B). Further, the levels of cytokines that activate ILC2 cells, such as IL-25 and IL-33, were similar in *Plscr1^–/–^CRTH2^–/–^* double–mutant mice and *Plscr1^–/–^* mice (Supplemental Figure 3, A and B), suggesting that the absence of CRTH2 did not alter these epithelial signals that recruit and/or activate ILC2s. Instead, defective ILC2 accumulation and diminished Type 2 immune responses in the absence of CRTH2 were regulated via ILC2 intrinsic mechanisms. We then isolated these ILC2 cells by flow sorting and found that ILC2s from various groups expressed similar levels of Gata3, suggesting ILC2 development was not affected by the absence of CRTH2 (Supplemental Figure 3, C and D). Importantly, we noticed that ILC2s that lacked both Plscr1 and CRTH2 had much lower levels of IL-25R and IL-33R/ST2 expression than *Plscr1^–/–^* ILC2s (Figure 6, C and D). In addition, IL-13 and IL-5 mRNA expression and protein secretion from ILC2s isolated from HDM-challenged *Plscr1^–/–^CRTH2^–/–^* double–mutant mice was much lower than ILC2s isolated from HDM-challenged *Plscr1^–/–^* mice (Figure 6, E–H). These studies demonstrate that CRTH2 played a key role in the effects of Plscr1 on ILC2 and IT2IR because the exaggerated Type 2 inflammation in HDM-treated *Plscr1^–/–^* mice was eliminated in *Plscr1^–/–^CRTH2^–/–^* double–mutant animals.

### Plscr1-null ILC2s have increased CRTH2 expression and signaling and are hyperactive in response to IL-33 treatment via CRTH2-dependent mechanisms.

**Figure 7:**
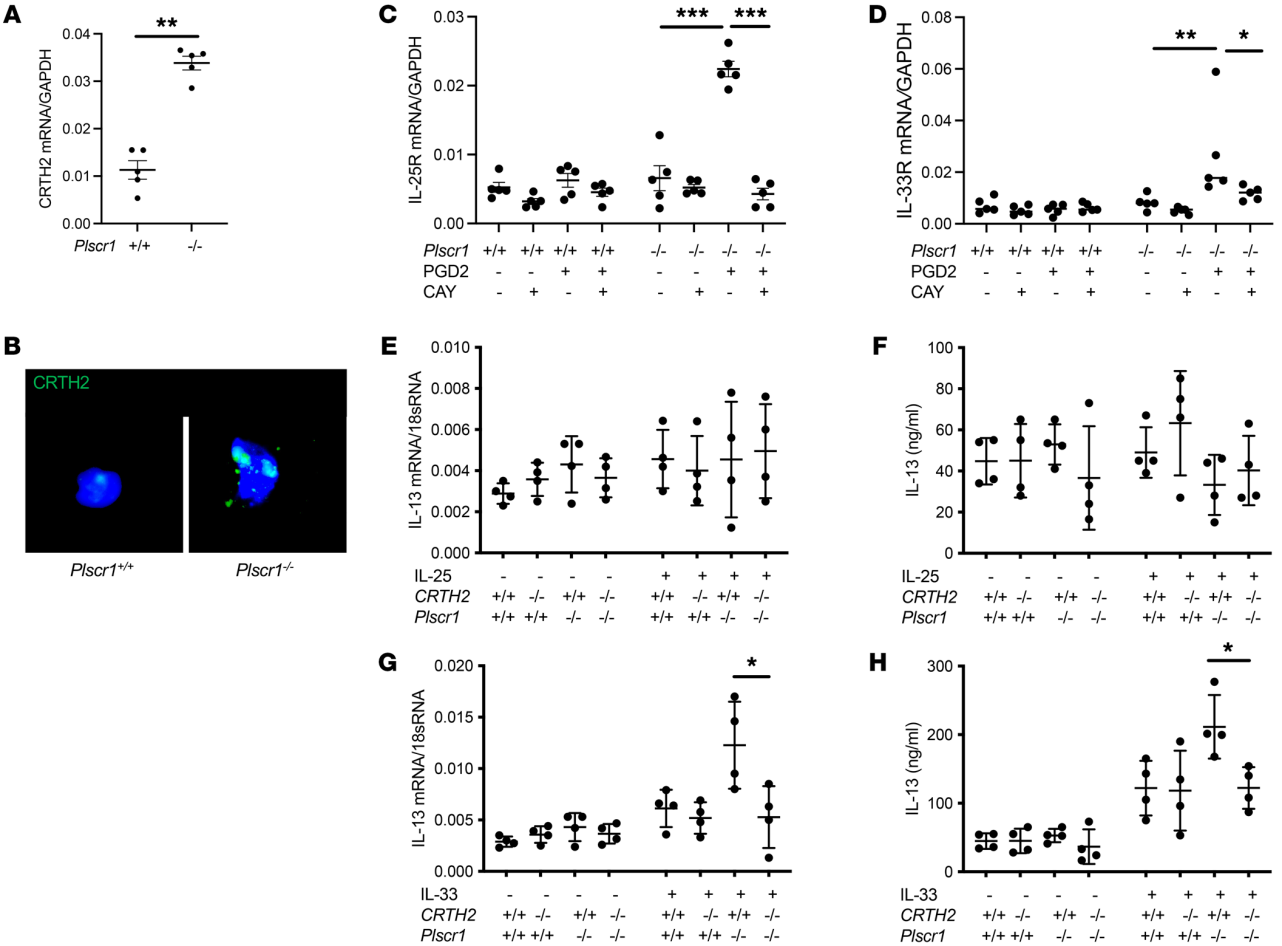
Plscr1-null ILC2s have increased CRTH2 expression and signaling, and are hyperactive in response to IL-33 treatment. Lung ILC2s were sorted from *WT* and *Plscr1^–/–^* mice. (**A**) CRTH2 mRNA were assessed by RT-PCR. (**B**) CRTH2 immunostaining was performed. (**C** and **D**) Sorted ILC2s were cultured in RPMI1640 supplemented with 10% FBS and a combination of IL-2 and IL-7, stimulated with PGD2 (200 nmol/L) in the presence or absence of CAY10471 (CAY, 1μM). IL-25R (**C**) and IL-33R (**D**) mRNA were assessed by RT-PCR. In **E** and **F**, lung ILC2s were sorted from *WT, CRTH2^–/–^, Plscr1^–/–^*, and *CRTH2^–/–^Plscr1^–/–^* mice. ILC2s were plated at 5–6 × 10^3^ cells/100 μL in 96-well plates. Sorted ILC2s were cultured in RPMI1640 supplemented with 10% FBS and stimulated with a combination of IL-2 and IL-7 with or without either IL-25 or IL-33 (10 μg/mL). IL-13 mRNA (**E** and **G**) and protein levels (**F** and **H**) were assessed by RT-PCR or ELISA. Values are mean± SEM with a minimum of 4 samples in each group. Comparisons between groups were conducted by 2-way ANOVA with Bonferroni’s post test. *P ≤ 0.05,**P ≤ 0.01,***P ≤ 0.001.

To further examine the expression of CRTH2 in ILC2s, lung ILC2s were sorted from *WT* and *Plscr1^–/–^* mice. We found that ILC2s isolated from *Plscr1^–/–^* mice had increased CRTH2 mRNA and protein expression (Figure 7, A and B). Sorted ILC2s were then cultured in RPMI1640 supplemented with 10% FBS with a combination of IL-2 and IL-7 and stimulated with PGD2 to activate CRTH2 signaling in the presence or absence of CRTH2 inhibitor CAY10471. We found that ILC2s isolated from *Plscr1^–/–^* mice had increased CRTH2 signaling in response to PGD2 stimulation by expressing higher levels of IL-25R and IL-33R (Figure 7, C and D). Importantly, the increased expression of IL-25R and IL-33R were completely blocked by CRTH2 inhibition (Figure 7, C and D).

To further investigate the effect of Plscr1 and CRTH2 on ILC2 activation, lung ILC2s were sorted from *WT*, *CRTH2^–/–^*, *Plscr1^–/–^*, and *CRTH2^–/–^Plscr1^–/–^* mice. Sorted ILC2s were then cultured in RPMI1640 supplemented with 10% FBS and stimulated with a combination of IL-2 and IL-7 with or without either IL-25 or IL-33 (10 μg/mL). We measured IL-13 transcription and secretion in the ILC2s to examine the activation status of these cells. We found that IL-25 did not directly activate ILC2s, as IL-13 levels were not altered after the treatment. This is consistent with the known function of IL-25, in that it mainly acts as an ILC2 chemoattractant (Figure 7, E and F). In contrast, IL-33 significantly activated IL-13 expression and secretion from ILC2s, and ILC2s from *Plscr1^–/–^* mice were hyperactive in response to IL-33 treatment (Figure 7, G and H). Importantly, we found that this hyperactive response was significantly diminished in cells that also lacked CRTH2 (Figure 7, G and H), suggesting that Plscr1-CRTH2 interaction played a major role in ILC2 activation.

### Overexpression of Plscr1 decreases HDM-induced accumulation of ILC2 cells and innate type 2 immune responses in the lung.

**Figure 8:**
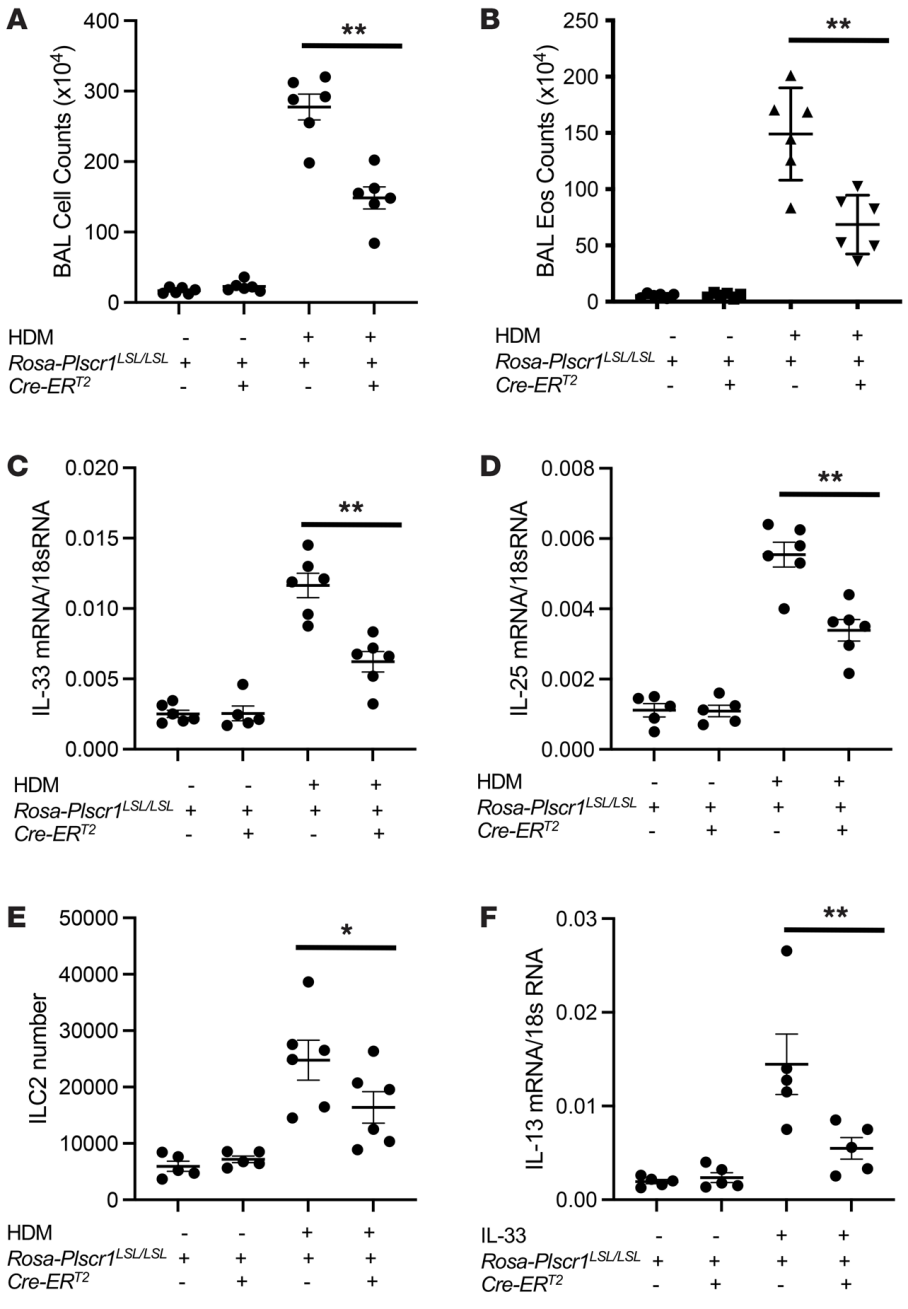
Overexpression of PLSCR1 decreases HDM-induced ILC2 cell accumulation and innate type 2 immune responses in the lung. Control and *Rosa-Plscr1^LSL/LSL^;Cre-ER^T2^* mice were subjected to HDM challenges. (**A** and **B**) BAL total cell and eosinophil counts were assessed by Diff-Quik staining. (**C** and **D**) Whole lung mRNA was extracted, and IL-33 mRNA (**C**) and IL-25 mRNA (**D**) were assessed by RT-PCR. (**E**) Lung ILC2 recovery was assessed by flow cytometry. (**F**) Isolated ILC2 cell mRNA was extracted, and IL-13 mRNA was assessed by RT-PCR. Values are mean± SEM with 4–7 mice in each group. Comparisons between groups were conducted by 2-way ANOVA with Bonferroni’s posthoc test. *P ≤ 0.05, **P ≤ 0.01.

We generated Rosa26 locus–targeted Plscr1 conditional knock-in transgenic mice (*Rosa26-loxP-STOP-LoxP-Plscr1 Tg; Rosa-Plscr1^LSL/LSL^*) that can be used to induce cell specific overexpression knock-in (KI) when crossed with cell-specific promoter-driven Cre mice (Supplemental Figure 4A). We confirmed Plscr1 upregulation in *Rosa-Plscr1^LSL/LSL^* mice when breeding with tamoxifen-inducible Cre (*Cre-ER^T2^*) mice (Supplemental Figure 4, B and C). Plscr1 was highly expressed by ILC2s (Supplemental Figure 4D) and its expression was not affected by HDM treatment (Supplemental Figure 4E). We then demonstrated that overexpression of Plscr1 decreased HDM-induced accumulation of ILC2 cells and innate type 2 immune responses in the lung, because BAL inflammation, eosinophilia, IL-33 and IL-25 expression, and ILC2 accumulation were all decreased in *Rosa-Plscr1^LSL/LSL^;Cre-ER^T2^* mice compared with Cre^–^ littermate controls (Figure 8, A–E). Consistently, ILC2s isolated from *Rosa-Plscr1^LSL/LSL^;Cre-ER^T2^* mice were hypoactive, in terms of IL-13 expression, in response to IL-33 treatment in vitro (Figure 8F). These results are consistent with previous findings that Plscr1 is a negative regulator of ILC2 activation and IT2IR.

### ILC2-specific overexpression of Plscr1 is sufficient to decrease HDM-induced accumulation of ILC2s and innate type 2 immune responses in the lung.

**Figure 9:**
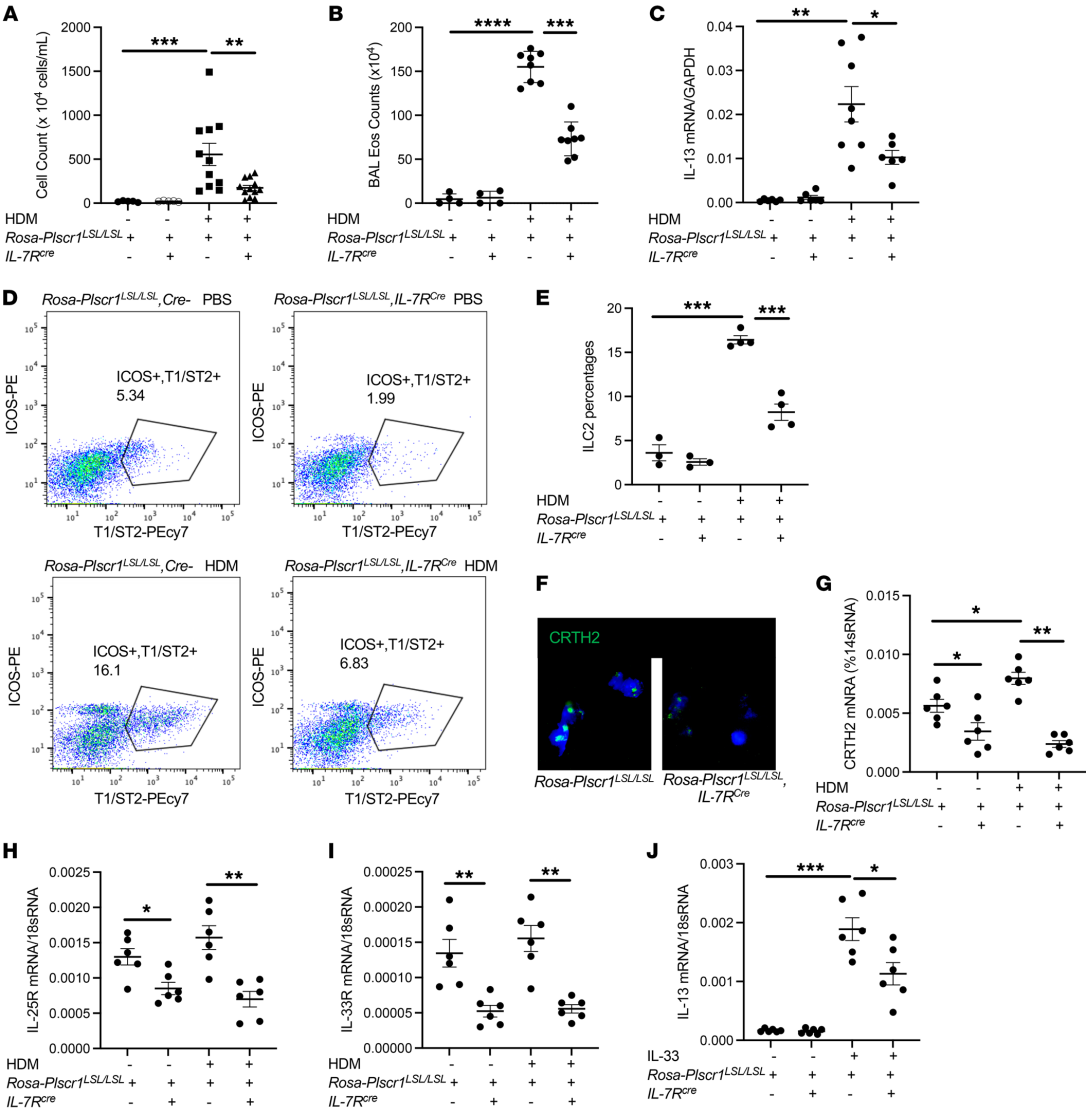
Overexpression of PLSCR1 in ILC2 cells decreases HDM-induced ILC2 cell accumulation and innate type 2 immune responses in the lung. *Rosa-Plscr1^LSL/LSL^* mice were bred with *IL-7R^cre^* mice to generate ILC2-specific Plscr1 overexpression. Cre^–^ and *Rosa-Plscr1^LSL/LSL^;IL-7R^cre^* mice were subjected to HDM challenges. (**A** and **B**) BAL total cell and eosinophil (Eos) counts were assessed by Diff-Quik staining. (**C**) Whole lung mRNA was extracted, and IL-13 mRNA was assessed by RT-PCR. (**D** and **E**) Lung ILC2 recovery was assessed by flow cytometry. (**F**–**I**) Lung ILC2s were sorted, CRTH2 immunostaining was performed (**F**), and CRTH2 (**G**), IL-25R (**H**), and IL-33R (**I**) mRNA were assessed by RT-PCR. (**J**) Sorted ILC2s were cultured in RPMI1640 supplemented with 10% FBS and stimulated with a combination of IL-2 and IL-7 with or without IL-33 (10 μg/mL). IL-13 mRNA was assessed by RT-PCR. Values are mean± SEM with 3–8 mice in each group. Comparisons between groups were conducted by 2-way ANOVA with Bonferroni’s posthoc test. *P ≤ 0.05, **P ≤ 0.01, ***P ≤ 0.001.

To investigate the specific role of Plscr1 on ILC2 cells, we bred *Rosa-Plscr1^LSL/LSL^* mice with the *IL7-R^cre^* mice, in which Plscr1 was specifically overexpressed only in the ILC2 population (28, 29). We then challenged these mice and the Cre^–^ controls with HDM. We found that HDM-induced BAL inflammation (Figure 9A), eosinophilia (Figure 9B), and lung IL-13 expression (Figure 9C) were decreased in *Rosa-Plscr1^LSL/LSL^;IL-7R^Cre^* mice compared with Cre^–^ controls. Importantly, the accumulation of ILC2 cells in the lung was also significantly decreased in *Rosa-Plscr1^LSL/LSL^;IL-7R^Cre^* mice (Figure 9, D and E). Consistently, ILC2s isolated from *Rosa-Plscr1^LSL/LSL^;IL-7R^Cre^* mice had decreased CRTH2 expression (Figure 9, F and G), decreased IL-25R and IL-33R expression (Figure 9H and I), and impaired response to IL-33 stimulation (Figure 9J). These results demonstrate that Plscr1 expression on ILC2s alone was sufficient to inhibit ILC2 activation and IT2IR.

## Discussion

Type 2 immunity is believed to have evolved to kill and expel metazoan parasites (helminths) and drive tissue repair (30). However, exaggerated, misdirected and/or prolonged type 2 immune responses also contribute to the pathogenesis of diseases like allergy, asthma, and pathological fibrosis (31). In keeping with the importance of these responses, research has focused on events that lead to adaptive Type 2 responses, Th2 lymphocyte polarization (32–35), and the Th2-effector responses induced by cytokines such as IL-4, IL-5, and IL-13 (36–38). However, more recent studies have made it clear that Th2 cells are not the only perpetrators of type 2 responses. In fact, these studies have highlighted the importance of IT2IRs and ILC2s in these disorders (25–27, 39–42). ILC2s are a family of innate lymphocytes that are distinct from adaptive immune cells such as T and B cells (41–43). ILC2s are identified in lung epithelial compartments, and they are able to rapidly produce large amounts of Type 2 cytokines and growth factors such as IL-5, IL-13, and amphiregulin (44–46). ILC2s are activated by epithelial cell-derived cytokines such as IL-25, IL-33, and TSLP, as well as other soluble factors such as PGD2 and LTD4 (25–27, 39, 40). ILC2s play a significant role in the pathogenesis of allergic asthma where they coordinate lung epithelial cells, structural cells and other innate and adaptive immune cells (41, 42). In doing so, ILC2s contribute to the initiation and the maintenance of the Type 2 immune responses by inducing eosinophilic lung inflammation, airway hyperresponsiveness, and mucus hypersecretion (47, 48). In addition, ILC2s stimulate M2 macrophage activation, skew dendritic cells toward a pro-Th2 phenotype, and enhance CD4^+^ T cell proliferation (49–51). They also enhance the production of amphiregulin, which drives healing and fibrotic repair (52). Our current study demonstrated that ILC2s are upregulated in response to HDM challenge, and high levels of IL-5 and IL-13 are produced by these cells. They also showed that Plscr1 was inhibited by IL-13. Further studies are needed to define other cytokines and mediators that regulate the expression of Plscr1 and are released from ILC2s, as well as the interaction between ILC2s versus other innate and adaptive immune cell types.

The mechanisms that contribute to the generation/accumulation of ILC2 cells and IT2IR, and the cell intrinsic factors that regulate ILC2 plasticity, differentiation, and function have not been adequately defined. PLSCR1 is the most studied member of the phospholipid scramblase protein family. It is a type II transmembrane protein whose main function is the bidirectional and nonspecific translocation of phospholipids between the inner and outer leaflets of the plasma membrane in response to Ca2^+^ mobilization (53–57). Our data demonstrated that null mutations of murine Plscr1 augmented lung ILC2 cell accumulation and IT2IR, and these augmented responses may be caused by higher levels of IL-25R and IL-33R/ST2 expression on ILC2 cells from Plscr1-null mice. On the other hand, Plscr1 expression on ILC2s alone is sufficient to decrease CRTH2 expression, decrease IL-25R and IL-33R expression, and inhibit ILC2 activation and IT2IR. ILC2s are delineated into at least 2 subsets: natural ILC2 (nILC2) and inflammatory ILC2 (iILC2) (58). nILC2s respond to IL-33 and are recognized by their expression of the IL-33 receptor (IL-33R, also known as ST2). iILC2s respond to IL-25 stimulation, migrate to inflammatory mucosal sites, and they express activation marker KLRG1 and IL-25R (59, 60). It is proposed that iILC2s are highly responsive precursors that are mobilized by inflammatory stimuli but ultimately adopt an nILC2-like or ILC3-like phenotype (60). ILC2s are typically characterized by their expression of Gata3, Hes1, Areg, Il5, and Il13 transcripts. However, transcriptionally distinct ILC2 subsets have been observed in various studies. For example, in the gut, 4 different subclasses of ILC2s are delineated based on their graded expression of Gata3, Klf4, Llrg1, Ly6a, Il2ra, and Areg (61). A subclass of ILC2, ILC1/2, also express Gzma, Hopx, and Epas1 normally associated with ILC1; while ILC2/3 produce Cxcl2, Cxcl3, and Arg1 characteristic of ILC3 (62). In the skin, a major ILC2 subset responding to IL-18 activation was identified (63). In the lung, 3 subsets of ILC2s were identified, ILC2a, ILC2b, and ILC2c, based on their expression of ILC2 signatures Gata3, Il1rl1, Klrg1m, Areg, Il5, Il13, Klrg1, and Pdcd1 (64, 65). Further analyses to define ILC2 subclasses and the plasticity possibly regulated by PLSCR1 are needed.

CRTH2 is a G-protein-coupled receptor known for binding with PGD2, is expressed by many immune cells, and is commonly used to identify ILC2 cells (25–27). We discovered the interaction between PLSCR1 and CRTH2 in yeast-2-hybrid assays using CRTH2 as the bait. These studies identified 12 clones that produced CRTH2 binding proteins. Interestingly, 11 of the 12 clones encoded Plscr1. The interactions between PLSCR1 and CRTH2 were verified by co-IP assays. We then generated *Plscr1^–/–^CRTH2^–/–^* double mutant mice to evaluate the role of CRTH2 in exaggerated ILC2 cell accumulation and IT2IR in *Plscr1^–/–^* mice, and we found that the exaggerated HDM-induced Type 2 inflammation in *Plscr1^–/–^* mice were significantly decreased in the absence of CRTH2. Higher levels of IL-25R and IL-33R/ST2 expression on ILC2 cells from Plscr1-null mice were diminished in cells recovered from *Plscr1^–/–^CRTH2^–/–^* double–mutant mice. Indeed, IL-13 production in response to IL-33 stimulation from ILC2s isolated from *Plscr1^–/–^CRTH2^–/–^* double–mutant mice were much lower than ILC2s isolated from *Plscr1^–/–^* mice, suggesting that ILC2s isolated from *Plscr1^–/–^CRTH2^–/–^* double–mutant mice are no longer hyperactive. It is important to note that the levels of cytokines that activate ILC2 cells, such as IL-25 and IL-33, were similar in *Plscr1^–/–^CRTH2^–/–^* double–mutant mice compared with *Plscr1^–/–^* mice. These findings suggest that CRTH2 depletion did not affect the secretion of IL-25 and IL-33 from epithelial cells. Instead, CRTH2-null ILC2s are intrinsically defective in response to IL-25 and/or IL-33 stimulation and activation. All together, these studies demonstrate that PLSCR1 interacts with CRTH2 in vivo and in vitro, and Plscr1 inhibits ILC2 activation via CRTH2-dependent mechanisms that modulate the expression of IL-25R and IL-33R. Consistently, using the Rosa26 locus–targeted Plscr1 conditional KI transgenic mice, we demonstrated that ILC2-specific overexpression of Plscr1 was sufficient to decrease HDM-induced accumulation of ILC2s and innate type 2 immune responses in the lung. It is worth mentioning that not all ILC2 are CRTH2^+^, and populations of CRTH2^–^IL7Rα^+^ and CRTH2^–^IL7Rα^–^ nonconventional ILC2s were identified in human blood and lung that are able to produce large amount of Type 2 cytokines (66). Nonetheless, these findings demonstrate that at least a subpopulation of ILC2s from lungs from Plscr1-null mice were primed to be activated. Further studies are needed to understand how member scramblase activity regulates cell surface receptor expression at transcriptional levels.

High levels of CRTH2 expression have been found in the lungs of patients with severe, uncontrolled asthma where they correlate with the levels of blood eosinophils (67, 68). Th2 cells express high levels of CRTH2 and migrate toward PGD2 in vitro. This chemotaxis is blocked by CRTH2 antagonism (69–71). In addition, PGD2-CRTH2 interaction is involved in the recruitment and activation of eosinophils and basophils (72, 73) and regulates the accumulation of ILC2s and helminth-induced Type 2 inflammation, suggesting that CRTH2 plays an important role in IT2IR in the lung (23, 24). As a result, clinical trials were designed to assess the effects of CRTH2 antagonism on lung function and asthma control. Surprisingly, orally administered CRTH2 antagonists have yet to demonstrate impressive efficacy in this disorder (74, 75). Most recently, fevipiprant failed to meet the primary endpoints in 2 phase III asthma trials (76), even though it has shown promising results in multiple phase II trials (77–79) and consistent reductions in asthma exacerbations rates were observed (80). It is interesting to speculate that these failures might be related to the dysregulation of CRTH2 binding partners, and CRTH2 antagonists may be more effective when given in combination with therapies targeting these partners, including PLSCR1. Direct measurement of phospholipid scramblase activity can be made possible by reconstituting PLSCR1 into large unilamellar liposomes composed of phophatidylcholine (PC), phophatidylglycerol, and fluorescent NBD-labeled PC (81). The extent to which NBD-PC molecules located in the inner leaflet are able to access the outer leaflet where the fluorescence signaling is eliminated by dithionite will determine the activity of PLSCR1. Based on our findings, it is reasonable to speculate that high-throughput assays to screen for the PLSCR1 agonists can be developed, and PLSCR1 agonists will be promising therapeutic options to treat Type 2 inflammation and asthma. One can also envision the development of effective combination therapies because CRTH2 antagonists may very well act in an additive or synergistic manner with interventions that augment the expression and/or activity of PLSCR1.

Most asthma starts from childhood in relation to sensitization to common inhaled allergens, such as house dust mites (HDM), cockroaches, animal dander, fungi, and pollens (82). Interestingly, epidemiologic and murine studies have also led to the belief that, in many patients and settings, asthma and asthmatic inflammation start in early life and can lead to lifelong affliction (83–85). A variety of lines of research suggest that exaggerated type 2 inflammatory responses contribute to atopic march, in which allergic sensitization early in life manifesting as eczema or atopic dermatitis would often progress to atopic rhinitis, and eventually to allergic asthma. Surprisingly, the mechanism(s) that underlie the chronicity of these responses and the degree to which they relate to Th2 versus ILC2 responses have not been defined. Our data demonstrate that the expression of Plscr1 is inhibited by IL-13 and Type 2 inflammation triggered by HDM. When combining with the studies that null mutations of murine Plscr1 augment lung innate Type 2 immune responses, these findings allow for the speculation that the inhibition of Plscr1 generates a positive feedback loop, which further augments ILC2 accumulation and IT2IR. Further proof-of-concept studies are needed to test the hypothesis that patients who develop exaggerated IT2IR as a result of PLSCR1 inhibition will go on to have more severe and persistent asthma symptoms.

For example, it would be important to compare the chimera mice receiving HDM-sensitized ILC2s from WT and Plscr1-null mice with mice receiving ILC2s isolated from *Rosa-Plscr1^LSL/LSL^;IL-7R^cre^* mice. A mouse atopic dermatitis model with type 2 inflammatory responses in the lung, or a model combining oxazolone-induced (Ox-induced) atopic dermatitis and HDM-induced lung inflammation is likely needed to fully address this hypothesis in the future. Overall, our observations led to the overall conclusion that PLSCR1 is a critical regulator of IT2IR in the lung. These studies will provide critical insights into biology and disease pathogenesis and identify targets that can be manipulated in attempts to control IT2IR in chronic diseases such as asthma.

## Methods

### Animal models.

Male and female, sex-matched, 8-to-10 week old C57BL/6 mice (at least 4 mice/group/experiment, repeated at least 3 times) were used in the studies. In the OVA model, mice were sensitized by i.p. injection of 20 μg OVA (Sigma-Aldrich) and 4 mg of aluminum hydroxide (Alum) (Pierce Chemical Co.) on days 0 and 7. One week later, mice were exposed to aerosolized OVA once a day for 3 days and sacrificed 1 day after the last OVA challenge. In the HDM model, mice were anesthetized with isoflurane prior to intranasal (i.n.) administration of either 25 μl PBS (control) or 25 μl (1 mg/mL in PBS) of purified HDM, Dermatophagoides pteronyssinus (Der P1), extract (B82, NC9756554, Lot 390884, Endotoxin approximately 7,800 EU/mg of protein, Greer Laboratories) 3 times a week for 3 weeks and sacrificed 18–24 hours after last HDM exposure. Methacholine responsiveness and lung function was assessed with Scireq FlexiVent.

### KO and transgenic mice.

*IL-13* transgenic (Tg) mice were generated in our lab as described previously (86). CRTH2-null (*CRTH2^–/–^*) mice were provided by Masataka Nakamura (Tokyo Medical and Dental University, Tokyo, Japan). *Plscr1^–/–^* mice (*Plscr1^tm1a(EUCOMM)Hmgu^*) were obtained from eucomm_komp. *Plscr1^–/–^* mice exhibit increased startle reflex, shortened QRS complex duration, abnormal retina morphology, but no defects in steady-state hematopoiesis and development of immune system (87, 88). Rosa26 locus–targeted Plscr1 conditional KI transgenic mice (*Rosa26-loxP-STOP-LoxP-Plscr1 Tg; Rosa-Plscr1^LSL/LSL^*) were generated at Brown Mouse Transgenic and Gene Targeting Facility. *Rosa-Plscr1^LSL/LSL^* mice were then bred with *Cre-ER^T2^* (The Jackson Laboratory) mice to generate inducible Plscr1–overexpression mice. Both Cre^–^ littermate controls and *Rosa-Plscr1^LSL/LSL^;Cre-ER^T2^* mice were injected with Tamoxifen (75mg/kg body weight) for 5 consecutive days. After 48 hours after the final injection, mice were used in experiments. ILC2s depleted *RoR*α*^fl/fl^/IL-7R^cre^* mice are gift from Andrew Mckenzie at MRC Laboratory of Molecular Biology (Cambridge, United Kingdom) and Hans-Reimer Rodewald from German Cancer Research Center (Heidelberg, Germany). *Rosa-Plscr1^LSL/LSL^* mice were also bred with *IL-7R^cre^* mice to generate ILC2-specific Plscr1 overexpression mice. All mice were congenic on a C57BL/6J background and were genotyped as previously described. Five generations of backcrosses were performed to generate the double mutant or multiply transgenic lines. Cre^–^ littermate controls were used to study *Rosa-Plscr1^LSL/LSL^* mice when breeding with *Cre-ER^T2^* mice or *IL-7R^cre^* mice.

### Histologic analysis.

Mouse lungs were removed en bloc, inflated to 25 cm pressure with PBS containing 0.5% low melting-point agarose gel, fixed, embedded in paraffin, sectioned, and stained with H&E. BAL and lung inflammation were assessed as described previously (89).

### Co-IP and Western blots.

Proteins from the lung lysate of *WT* mice, *IL-13* Tg mice, and primary mouse peritoneal macrophages treated with or without IL-13 were clarified by centrifugation at 15,000*g* for 10 minutes at 4**°** C. Plscr1 protein level was then evaluated by Western immunoblot as previously described (90, 91). In separate experiments, proteins from the lung lysate of *WT* mice, *IL-13* Tg mice, and HEK293 cells that were transfected with CRTH2 or Plscr1 were immunoprecipitated with anti-CRTH2 (Santa Cruz, sc-271898; or ABBiotec, 253687) or rabbit anti-mouse Plscr1 monoclonal antibody (Proteintech, 11582-1-AP), respectively, using Catch and Release V2.0 (Reversible Immunoprecipitation System, Sigma-Aldrich). The precipitates were subjected to immunoblotting with antibodies against CRTH2 or Plscr1, respectively. Western blots for signaling were performed as previously described (90, 91).

### In vivo IL-33 silencing.

According to the established method in our laboratory (26, 27), control or HDM-treated *WT* and *Plscr1^–/–^* mice were randomized to receive IL-33-specific or scrambled siRNA i.n. 3 times a week (2 nmol/mouse/day) starting on Day 4.

### Immunofluorescence staining.

To localize the expression of Plscr1 in various cell populations, lungs from *WT* mice with or without HDM challenges were sectioned, SPC (Santa Cruz, 13979), CX3CR1 (Thermo Fisher Scientific, 14-6093-81), or Gata-3 (Abcam, 199428) was labeled with red fluorescence (Alexa Fluor 594) and Plscr1 (Invitrogen, MA5-19636) was labeled with green fluorescence (Alexa Fluor 488). To localize the expression of Plscr1 and CRTH2, double-label immunofluorescence staining was undertaken using Paraffin-embedded lungs from *WT* and *IL-13* Tg mice. Monoclonal anti-Plscr1 (Proteintech, 11582-1-AP), and anti-CRTH2 (Santa Cruz, sc-271898) were used in these evaluations. CRTH2 was labeled with red fluorescence (Alexa Fluor 594) and Plscr1 was labeled with green fluorescence (Alexa Fluor 488).

### Flow cytometry.

APC-conjugated antilineage markers, PE-conjugated anti-ICOS, FITC-conjugated anti-CD90.2, and PE-Cy7–conjugated anti-T1/ST2 were obtained from BD Pharmingen (no. 558074) and eBioscience (no. 12-9949-81, no. 11-0903-82, and no. 25-9335-82). Flow cytometry was performed using BD FACSAria. ILC2 are defined as Lin(CD3, CD11b, CD45R/B220, Ly-76, Ly6G, and Ly-6C)-, Thy1.2^+^ICOS^+^T1/ST2^+^ cells. Data were analyzed using FlowJo software v.10 (TreeStar Inc.). Percentages of Lin-Thy1.2^+^ICOS^+^T1/ST2^+^ cells and total cells recovered from the lungs were used to determine the number of ILC2 cell recovery. For all analyses, isotype control staining was subtracted from true antibody staining to determine the percentage of positive cells.

### ILC2 amplification and adoptive cell transfer.

*WT* and *Plscr1^–/–^* mice were treated with 1 μg IL-33 i.n. daily for 5 consecutive days to allow in vivo ILC2 amplification and lung ILC2s were harvested as described above. 5 × 10^5^ ILC2s were inoculated i.n. to *RoR*α*^fl/fl^/IL-7R^cre^* mice. Mice were challenged with HDM 1 day after ILC2 adoptive transfer.

### ILC2 culture.

Lung ILC2s were sorted from *WT*, *CRTH2^–/–^*, *Plscr1^–/–^*, *CRTH2^–/–^Plscr1^–/–^* mice, *Rosa-Plscr1^LSL/LSL^;Cre^–^* mice, and *Rosa-Plscr1^LSL/LSL^;IL-7R^Cre^* mice. ILC2s were plated at 5–6 × 10^3^ cells/100 μl in 96-well plates. Sorted ILC2s were cultured in RPMI1640 supplemented with 10% FBS and a combination of IL-2 and IL-7, stimulated with PGD2 (200 nmol/L) in this presence or absence of CAY10471 (CAY). In a separate experiment, sorted ILC2s were cultured in RPMI1640 supplemented with 10% FBS and stimulated with a combination of IL-2 and IL-7 with or without either IL-25 or IL-33 (10 μg/mL). Total RNA was then extracted and used for gene expression analyses.

### Gene expression analysis.

Cells processed from mouse lungs were lysed in TRIzol reagents and then total cellular RNA was extracted by Qiagen RNeasy kit per manufacturer’s instructions. From the mRNA, cDNA was synthesized using the BIORAD iScript cDNA Synthesis kit per manufacturer’s instructions. The corresponding mRNA level was then measured using real time PCR. The primer sequences for Plscr1, IL-25, IL-33, IL-25R, IL-33R, IL-13, IL-5, Gata-3, and CRTH2 were obtained from PrimerBank (http://pga.mgh.harvard.edu/primerbank/). The sequences are: Plscr1-F, GCCCAAGTTCACTCTCCAAA, Plscr1-R: GAGCTCAAAGTCAATGTCGG; IL33R-F, CCAGTAAGTGAGACAGCAGCATTT, IL33R-R, CTGTAGATACCCAGATGAAGGGCT; IL-25R(IL17RB)-F, GACGCGAAGGGACAGTTG, IL-25R(IL17RB)-R, CAGCAGCACCAGGAAGAGAG; CRTH2-F, CCTTTTTTCCACCTTGCCATG, CRTH2-R, CCAGGATAGTTGGCATGTC; 18sRNA-F, CGGCTACCACATCCAAGGAA, 18sRNA-R, GCTGGAATTACCGCGGCT; GAPDH-F, AGGTCGGTGTGAACGGATTTG, GAPDH-R, TGTAGACCATGTAGTTGAGGTCA; Rpl13a-F, AGGGGCAGGTTCTGGTATTG, Rpl13a-R, TGTTGATGCCTTCACAGCGT; IL-25-F: ACCACAACCAGACGGTCTTC, IL-25-R: TGTACACCTGGCCCTCTCTC; IL-33-F: GCTGCGTCTGTTGACACATT, IL-33-R: GACTTGCAGGACAGGGAGAC; GATA3-F: CTGGAGGAGGAACGCTAATG, GATA3-R: CAGGGATGACATGTGTCTGG; IL-5-F, TCAGGGGCTAGACATACTGAAG, IL5-R, CCAAGGAACTCTTGCAGGTAAT; and IL13-F, ATGCCCAACAAAGCAGAGAC, IL13-R, TGAGAGAACCAGGGAGCTGT.

### ELISA.

BAL IL-13, serum IgE, BAL SLPI, and soluble IL-33R/ST2 levels were assayed using commercially available ELISA kits (R&D) following the manufacturer’s instructions.

### Statistics.

Mouse data are expressed as mean ± SEM. As appropriate, groups were compared by 2-way ANOVA with Bonferroni’s posthoc test; follow-up comparisons between groups were conducted using a 2-tailed Student’s *t* test. *P* ≤ 0.05 was considered significant. Statistical analysis was performed using Graphpad (Graphpad Software Inc.). Graphs were generated using Excel and Graphpad.

### Study approval.

Animal experiments were approved by the Institutional Animal Care and Use Committee of Brown University in accordance with federal guidelines.

### Data availability.

Data are available in the supporting data values XLS file or from the corresponding author upon request.

## Author contributions

AHG, SM, AE, AC, and YZ designed the research study. AHG, SM, AE, AC, CHH, DY, and YZ conducted the experiments. AHG, SM, AE, AC, CHH, DY, and YZ acquired data. AHG, SM, AE, AC, and YZ analyzed data. PS, AXY, CN, CHH, CL, CGL, and JAE provided reagents. AHG and YZ wrote the manuscript.

## Supplementary Material

Supplemental data

Supporting data values

## Version 1 06/08/2023

In-Press Preview

## Version 2 08/01/2023

Electronic publication
