# Supplementary material for: Phospholipid scramblase-1 regulates innate type 2 inflammation in mouse lungs via CRTH2-dependent mechanisms
Source: J Clin Invest. 2023 Aug 1;133(15):e169583. doi: 10.1172/JCI169583 (PMC10378191; doi:10.1172/JCI169583)
Supplement: Supplemental data [file jci-133-169583-s122.pdf]

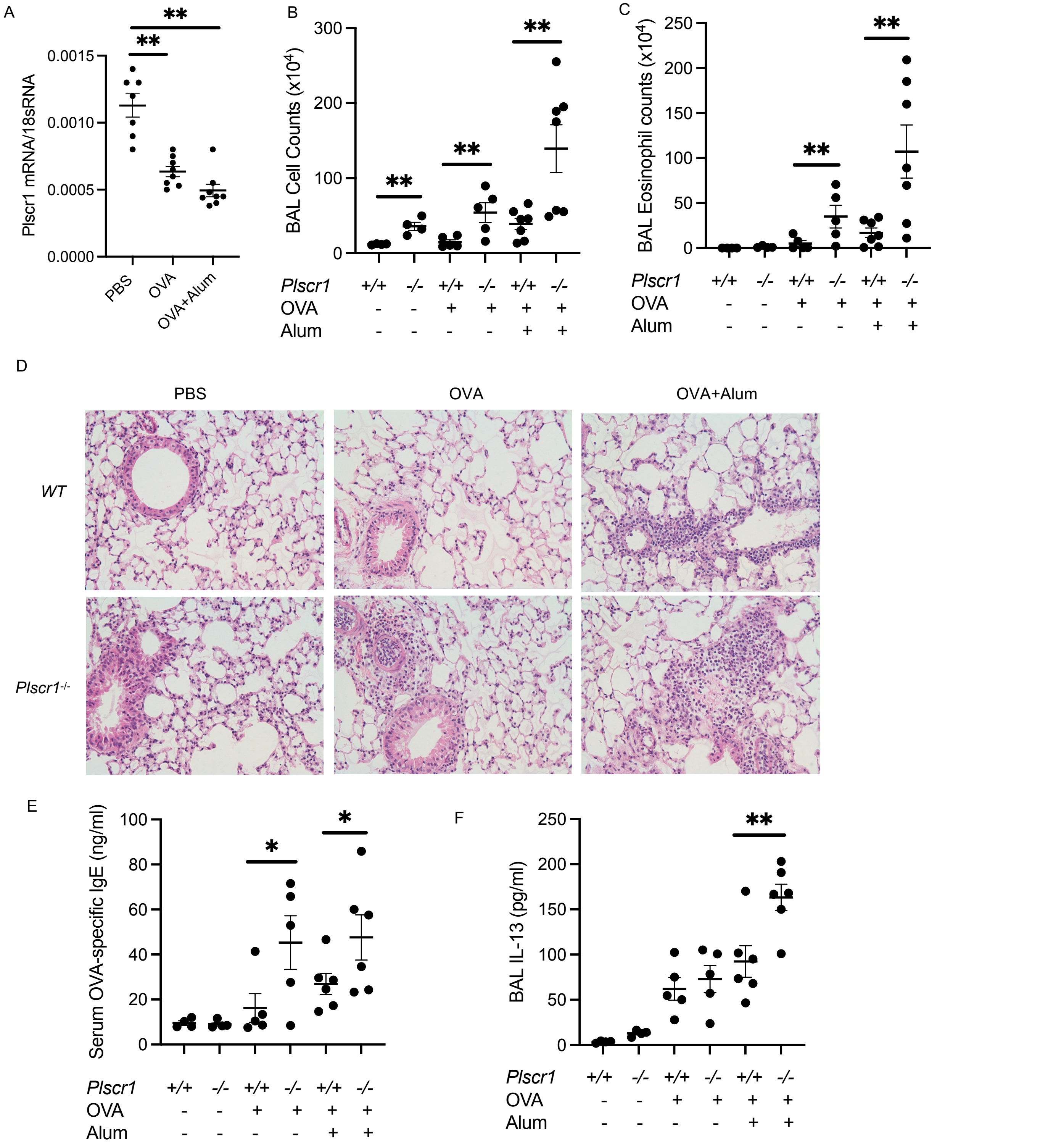

Supplemental Fig 1. Null mutations of Plscr1 significantly augments OVA-induced type 2 immune responses. (A) *WT* mice were subjected to OVA sensitization and challenge, with or without alum as the conjugant, lung Plscr1 mRNA expression was assessed by qRT-PCR. Values are mean± SEM with 7-9 mice in each group. Data was assessed with unpaired Student's t-test. **p ≤ 0.01. (B and C) *WT* and *Plscr1*$^{-/-}$ mice were subjected to OVA sensitization and challenge, with or without alum as the conjugant. BAL total cell and eosinophil counts were assessed by Diff-Quik staining. (D) H&E staining of *WT* and *Plscr1*$^{-/-}$ mice with and without OVA administration. (E) Serum OVA-specific IgE levels were measured by ELISA. (F) BAL IL-13 levels were quantitated by ELISA. Values are mean± SEM with 4-7 mice in each group. Comparisons between groups were conducted by two-way ANOVA with Bonferroni's post test. *p ≤ 0.05,**p ≤ 0.01.

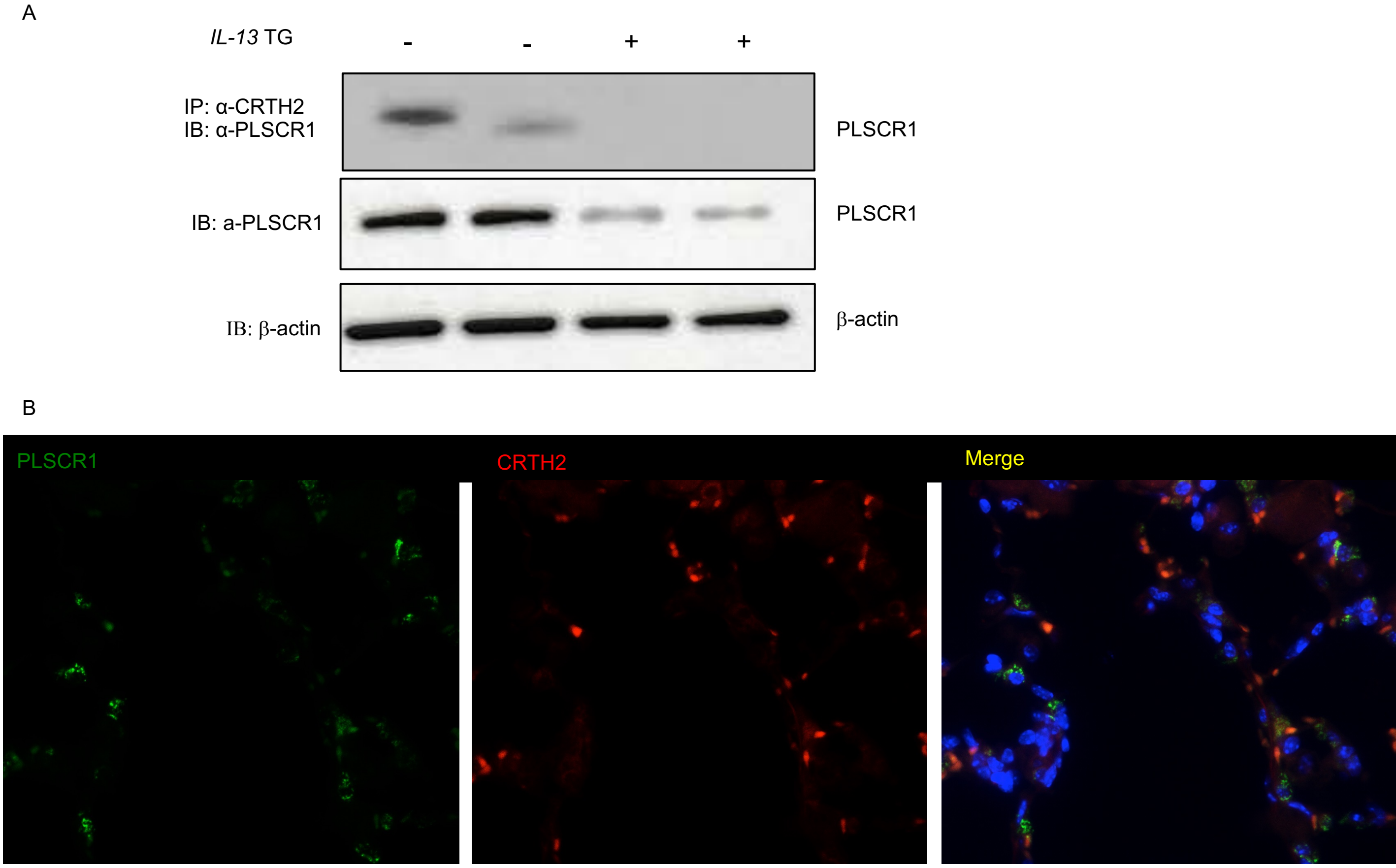

<u>Supplemental Fig 2.</u> The binding between PLSCR1 and CRTH2 in *IL-13* Tg lungs. (A) *WT* and *IL-13* Tg mouse lung protein lysates were co-immunoprecipitated (Co-IP) with α-CRTH2 antibody and immunoblot (IB) with α-PLSCR1. Individual IBs with α-PLSCR1 and actin were also included. (B) Lungs from HDM-treated mice were sectioned, CRTH2 was labeled with red fluorescence (alexafluor 594) and Plscr1 was labeled with green fluorescence (alexafluor 488). Co-localization of CRTH2 and Plscr1 is indicated by arrows. Nuclei are stained with DAPI (blue). Images are representative of 3 mice.

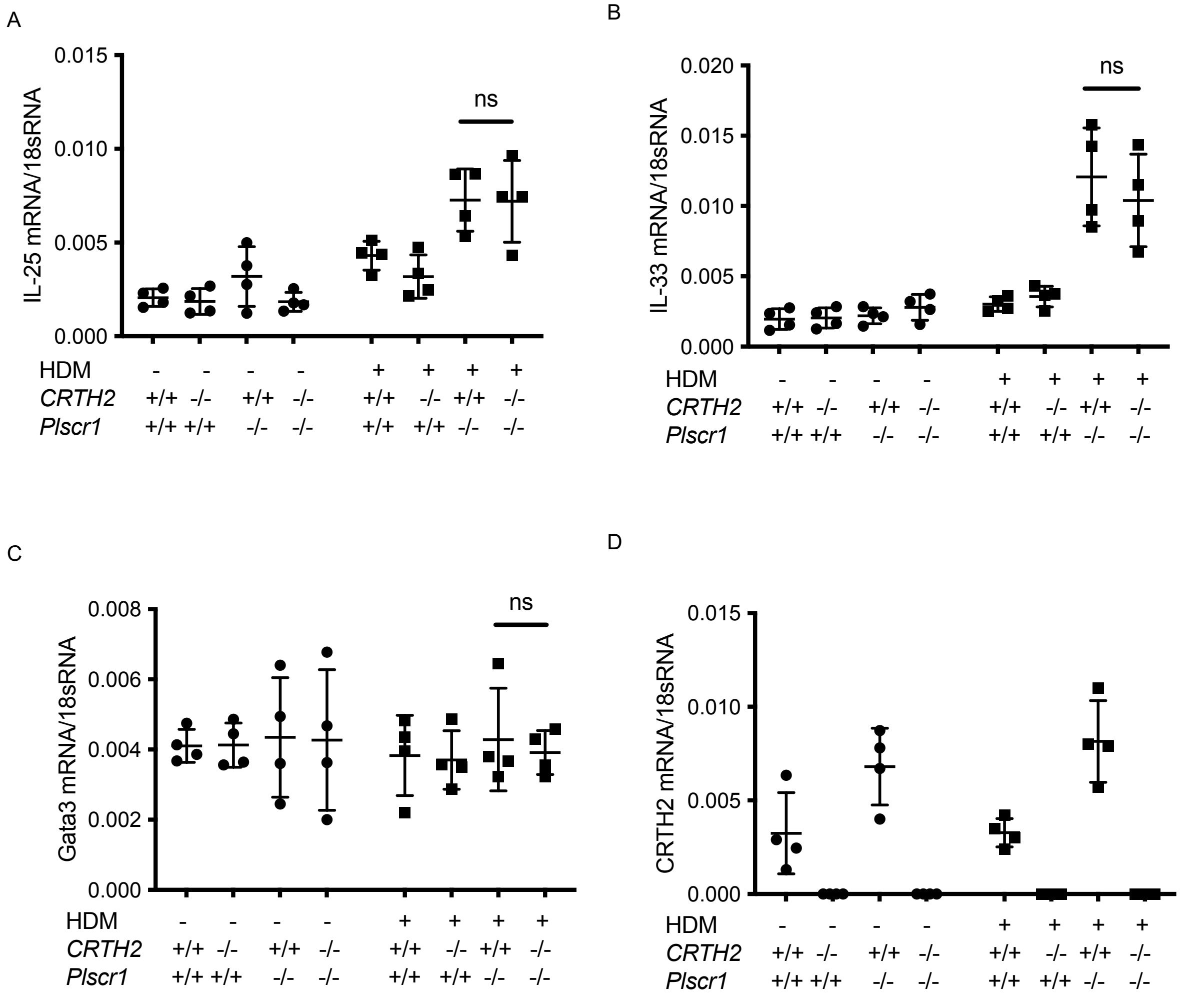

<u>Supplemental Fig 3.</u> **IL-25 and IL-33 secretion, and Gata3 expression were not affected by the absence of CRTH2.** *WT*, *CRTH2*$^{-/-}$, *Plscr1*$^{-/-}$, and *CRTH2*$^{-/-}$*Plscr1*$^{-/-}$ mice were subjected to HDM administration. In (A) and (B), whole lung mRNA was extracted and IL-25 and IL-33 mRNA levels were assessed by RT-PCR. In (C) and (D), ILC2 were sorted and mRNA was extracted from ILC2s, and Gata3 and CRTH2 mRNA were assessed by RT-PCR. Values are mean± SEM with a minimum of 4 mice in HDM group. Comparisons between groups were conducted by two-way ANOVA with Bonferroni's post test. NS, not significant.

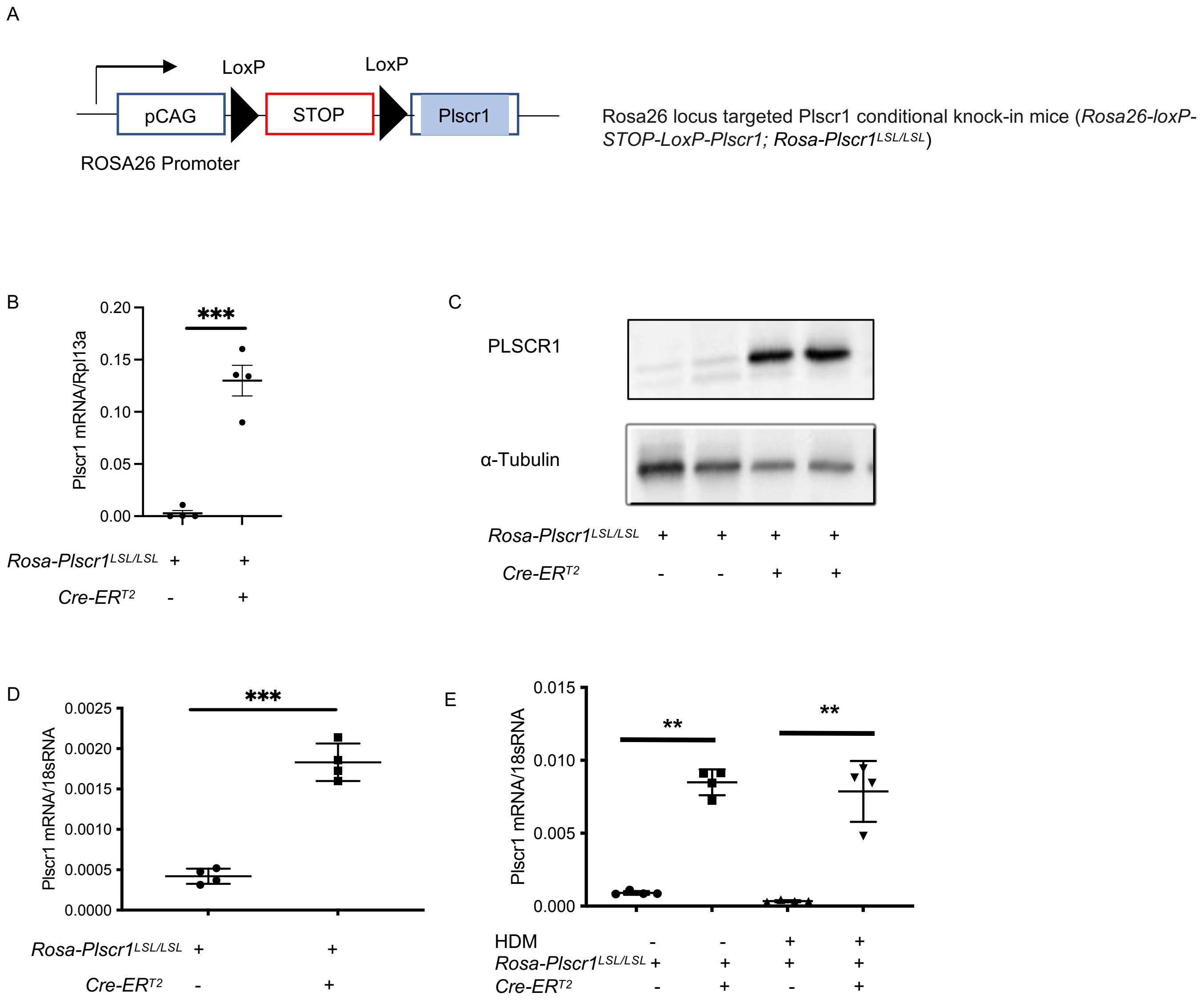

<u>Supplemental Fig 4.</u> Generation of conditional Rosa-Plscr1 transgenic knock-in mouse model. (A) Rosa26 locus targeted PLSCR1 conditional knock-in (KI) transgenic mice (*Rosa26-loxP-STOP-LoxP-Plscr1 Tg; Rosa-Plscr1*$^{LSL/LSL}$). (B) *Rosa-Plscr1*$^{LSL/LSL}$ mice were crossed with *Cre-ER*^{T2} mice, and were subjected to TAM injection to induce PLSCR1 expression, and PLSCR1 mRNA overexpression was assessed by qRT-PCR. (C) PLSCR1 protein level was evaluated by Western Immunoblot (IB) analysis. (D) Plscr1 mRNA expression was assessed by qRT-PCR in sorted ILC2 cell population. (E) *WT* and *Rosa-Plscr1*$^{LSL/LSL}$*;Cre-ER*^{T2} mice were subjected to TAM injection to induce PLSCR1 expression, challenged with or without HDM, and mRNA expression was assessed by qRT-PCR in whole lung RNA. Values are mean± SEM with a minimum of 4 mice in HDM group. Data was assessed with unpaired Student's t-test. Comparisons between groups were conducted by two-way ANOVA with Bonferroni's post test.
